# Sex differences in the physiological responses to cardiac rehabilitation: a systematic review

**DOI:** 10.1186/s13102-024-00867-9

**Published:** 2024-03-28

**Authors:** J. Bouakkar, T.J. Pereira, H. Johnston, M. Pakosh, J. D. M. Drake, H. Edgell

**Affiliations:** 1 School of Kinesiology and Health Science, Bethune College, York University, 4700 Keele St, Toronto, ON M3J 1P3 Canada; 2 University Health Network, Toronto, ON Canada; 3 Muscle Health Research Centre, York University, Toronto, ON Canada

**Keywords:** Cardiac rehabilitation, Sex differences, Physiology, Autonomic, Cardiovascular, Metabolic, Fitness, Body composition

## Abstract

**Background:**

Heart disease is one of the leading causes of death in Canada. Many heart disease patients are referred for cardiac rehabilitation, a multidisciplinary outpatient program often consisting of exercise training. Cardiac rehabilitation has been proven to be a successful secondary preventative measure in reducing mortality and improving overall health in heart disease patients, and its completion is important for both sexes as there is growing evidence that women benefit as much as men, if not more, with regard to mortality. It is important to note that previous studies have shown that healthy men and women respond differently to aerobic and resistance training, possibly due to hormones, body composition, autonomic and/or cardiovascular differences. However, evaluating sex differences in the efficacy of standard cardiac rehabilitation programs has not yet been fully explored with many studies investigating clinical or anthropometric data but not physiological outcomes. This systematic review aimed to investigate physiological differences in male and female heart disease patients after cardiac rehabilitation. The inclusion criteria were purposefully broad to encompass many cardiac rehabilitation scenarios, many cardiac disease states, and various program lengths and intensities with the intention of highlighting strengths and weaknesses of the current body of literature.

**Methods:**

To conduct a synthesis without meta-analysis, a search strategy was generated to examine the relationships between heart disease patients, a supervised exercise program, physiological outcomes, and sex differences. The review was registered (Prospero: CRD42021251614) and the following databases were searched from inception to 19 December 2023: APA PsycInfo (Ovid), CINAHL Complete (EBSCOhost), Embase (Ovid), Emcare Nursing (Ovid), Medline All (Ovid; includes PubMed non-Medline), and Web of Science Core Collection. Eighty-eight studies pertaining to fitness, metabolism, body composition, respiratory function, cardiac function and C-reactive protein underwent data extraction.

**Results and conclusions:**

Importantly, this review suggests that men and women respond similarly to a wide-range of cardiac rehabilitation programs in most physiological variables. However, many studies discussing maximal oxygen consumption, functional capacity, six-minute walk distances, and grip strength suggest that men benefit more. Further research is required to address certain limitations, such as appropriate statistical methods and type/intensity of exercise interventions.

**Supplementary Information:**

The online version contains supplementary material available at 10.1186/s13102-024-00867-9.

## Introduction

Heart disease (HD) is one of the leading causes of death in Canadian women with ischemic HD responsible for the vast majority at almost 14,000 deaths per year [1]. The age-standardized prevalence of ischemic HD in Canadian women is 6.3% compared to 10.0% in men [2], however, recent studies have shown that women are three times more likely to experience a major adverse cardiac event following catheterization compared to men after being diagnosed with stable angina and nonobstructive coronary artery disease (CAD) [3]. This is noteworthy considering the underrepresentation of women in clinical trials and the lack of research on sex-specific factors of cardiovascular disease in women, including prevention, diagnosis, clinical testing, treatment, and therapy [4].

Cardiac rehabilitation (CR) programs have been shown to be beneficial for both men and women with regard to mortality [5–7], yet significant sex differences exist in cardiovascular anatomy and physiological functioning across multiple areas of health and fitness research [8]. For example, sex-specific differences have been demonstrated when comparing men and women in terms of anatomical size, bodily composition, sympathetic or parasympathetic responses to stressors, and endocrine responses [9–11]. This disparity of physiological responses should be further investigated to help develop appropriate sex-specific medicine and interventions, particularly in light of evidence that men and women respond to exercise training along a different time course and to a different magnitude regarding cardiac dimensions and cardiorespiratory fitness [12]. Further, women who complete CR programs can improve mortality, however, they are less likely to be referred to CR and less likely to complete it [13–15]. Evidence of physiological improvements due to CR completion in women will serve to strengthen the body of evidence highlighting the benefits in women. This systematic review without meta-analysis aims to summarize pre-clinical evidence from research studies that examined the physiological responses to the completion of supervised CR exercise programs in men and women with all types of chronic HD. We aim to highlight the strengths and weaknesses of the work that has been completed to suggest improvements and future studies that need to be completed.

## Methods

### Data sources and search strategy

**Fig. 1:**
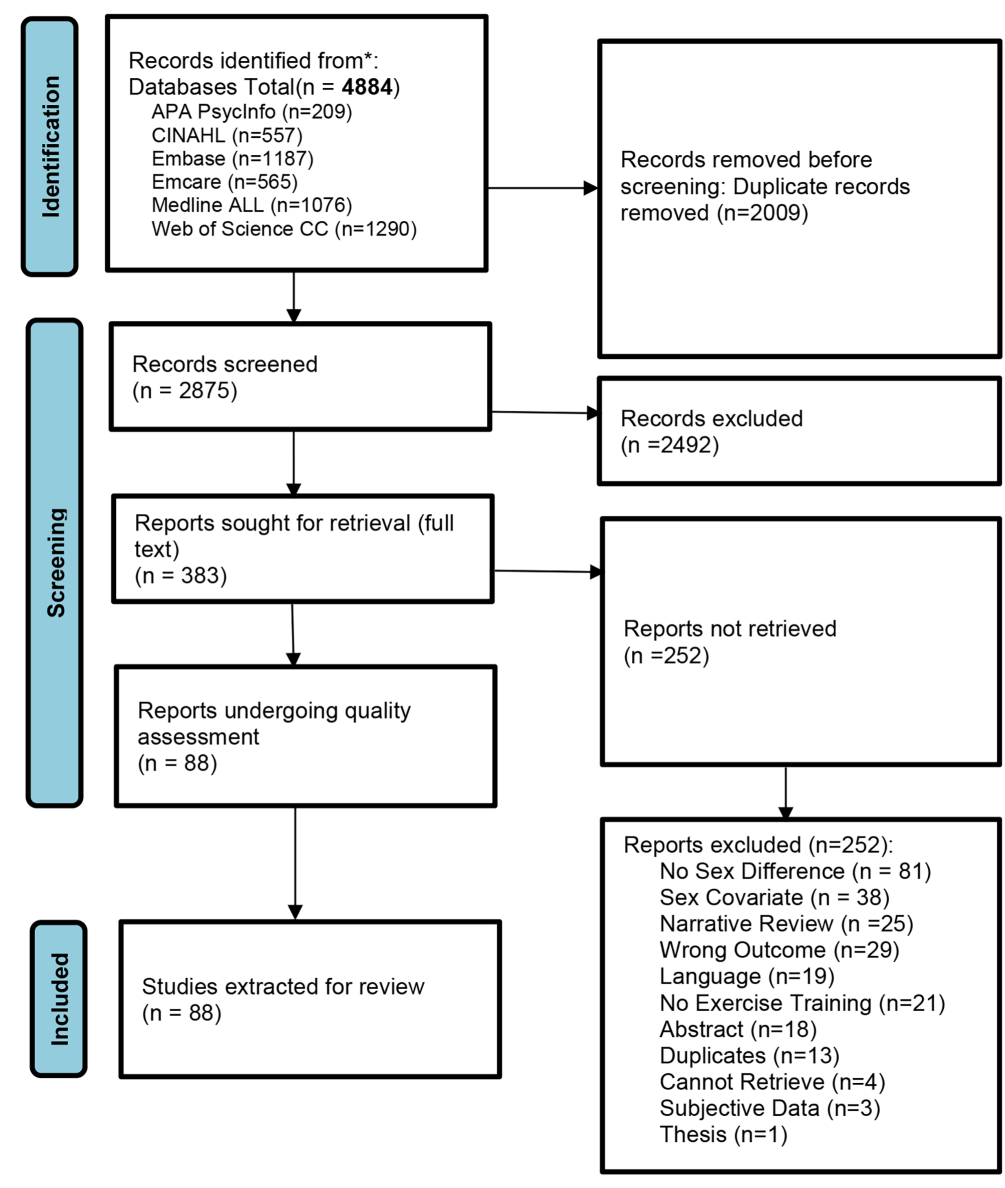
PRISMA flow diagram describing the search for relevant articles

This review was conducted in accordance with the Preferred Reporting Items Systematic Reviews and Meta-Analyses [PRISMA] guidelines [16] (Fig. 1); 4884 records were retrieved from six electronic databases.

The databases were searched from inception to 19 December 2023: APA PsycInfo (Ovid), CINAHL Complete (EBSCOhost), Embase (Ovid), Emcare Nursing (Ovid), Medline All (Ovid; includes PubMed non-Medline), and Web of Science Core Collection. The search was developed and finalized with the expertise of a librarian (MP) and utilized the PICO framework where all cardiac disease patients comprised the Population, cardiac/exercise rehabilitation was the Intervention, and gender/sex was the Comparator. To keep the results purposefully broad, no Outcome concept was added, as all physiological responses were of interest. No date or language limits were applied to the original search, but only English abstracts and articles were extracted. A hand search was conducted, but no additional articles were retrieved. The Medline search strategy is available in Supplemental Fig. 1.

### Prospero and covidence registration

The review underwent Prospero registration (CRD42021251614) and was analyzed using Covidence. The review team included the primary author (JB) and four other peer reviewers for screening (TP, HJ, JD, HE). Two reviewers and the primary author would agree on a final consensus if there was a discrepancy during screening stages. Duplicates were removed, reducing the number of citations from 4884 to 2875.

### Study selection and screening

Included studies focused on male and female patients with cardiac disease undergoing CR with supervised exercise. A sex difference needed to be directly investigated rather than as a covariate in the statistical model. The studies were original research studies, excluding books, abstracts, or case studies. Additionally, the methodology must have included quantitative measurements of physiological variables before and after the completion of CR. Qualitative research or research focusing on psychosocial aspects measured via questionnaires, while of great importance, were not the focus of the current investigation. A screening pilot on 25 articles was conducted to ensure consistency among reviewers.

The finalized criteria for title and abstract screening included cardiac/HD patients, an outpatient CR program which included a supervised exercise component (at-home programs with remote supervision were included), a physiological response before and after the program, and a comparison between men and women. A total of 383 articles out of 2875 titles/abstracts were included in the next stage. The full text of articles was retrieved using York University or University of Toronto Libraries. Screeners identified if there were physiological assessments at both baseline and CR completion; variables such as mortality or non-physiological variables were excluded. Sex differences were further screened at this stage. For example, many studies included both men and women and used covariate analysis for sex differences without disaggregation of the data; these studies were excluded. Lastly, any review with no original data on sex differences was also excluded. Only 88 out of the 383 progressed to the next round of screening. The JBI (Joanna Briggs Institute) quality assessment (QA) tool for cohort studies was used and modified to include an additional question regarding if empirical evidence was provided with sex difference conclusions. Two reviewers assessed the 88 papers gathered at the QA stage. No papers were eliminated based on a quality threshold.

## Results

88 final articles were extracted (Table 1). Only 32 physiological variables described by ≥ 3 articles were included in Table 2 and discussed in the current review; 118 physiological variables which were described by ≤ 2 articles are listed in Supplemental Table 1. Extracted information included the title of the article, authors, population description, total number of participants, type and length of CR program, QA score, adherence rates, and statistical modelling used (Table 1). After each extraction, the papers were quantified as to whether a sex difference was or was not present per variable (Table 2). A thematic synthesis was completed after assessing all the findings and data.

**Table 1:** Study characteristics

Study ID		Population description	Total number of participants in relevant cohort	Type of CR	Length of CR	Completion and/or Adherence (sex presented separately where available)	Quality Assessment (out of 12)	Statistics for relevant comparisons	
Adams 1999 [102]		Patients with CABG, MI, PTCA, cardiomyopathy, and stable angina	*n* = 61 (46 men, 15 women)	HIIT & Aerobic	8 Weeks	CR Completion 100%	11	ANOVA	
Ades 1992 [27]		All patients (hospitalized from AMI or CABG) interviewed were candidates for participation in cardiac rehabilitation	*n* = 57 (39 men, 18 women)	Aerobic	12 Weeks	CR Completion 95% 37/39 men, 94% 17/18 women	11	t-test	
Anjo 2014 [53]		Patients with CAD who attended a cardiac rehab program after an ACS or elective PCI	*n* = 386 (301 men, 85 women)	Aerobic and Strength	8–12 Weeks	> 80% sessions attended	11	t-test	
Antunes-Correa 2010 [28]		Patients with clinically stable HF, aged between 40 to 70 years, in NYHA functional class II to III and with an ejection fraction < 40%	*n* = 21 (12 men, 9 women)	Aerobic and Strength	16 Weeks	85–100% of sessions attended.	12	ANOVA	
Araya-Ramirez 2021 [64]		Cardiac patients enrolled in a university-based CR program	*n* = 311 (237 men, 74 women)	Aerobic	12 Weeks	92% adherence for men and women	12	ANCOVA	
Balady 1996 [54]		Patients experiencing MI, CABG, or PTCA within 6 months of program	*n* = 778 (558 men, 220 women)	Aerobic with Strength at some centers.	10 ± 2 weeks	CR completion: 33% 182/558 men, 28% 61/220 women; 83% adherence rate for men and women	12	t-test	
Baranyi 2022 [103]		Patients with acute MI	*n* = 56 (45 men, 11 women)	Not described	4 weeks	CR completion: 100%	10	ANCOVA	
Bellet 2015 [65]		Patients with a diagnosis of MI, angina, controlled arrhythmia, CAD or PCI, CABG, or experienced cardiac valve surgery, defibrillator, or pacemaker implantation. Patients were divided into a fast-track CR or a traditional CR program	*n* = 197 (130 men, 67 women) Fast Track CR, *n* = 423 (332 men, 91 women) Traditional CR	Aerobic and Strength	6 Weeks	CR completion: 58% 115/197 fast track CR patients and 60% 254/423 traditional CR patients.	11	ANOVA	
Braga 2021 [96]		Patients were diagnosed with ACS	*n* = 731 (633 men, 98 women)	Aerobic and Strength	Up to 12 Weeks	> 50% sessions attended	10	t-test	
Branco 2016 [55]		Patients diagnosed with CAD who have been referred to CR	*n* = 1399 (1068 men, 331 women)	Aerobic and Strength	8–12 Weeks	CR completion: 83% 886/1068 men, 72% 238/331 women	12	t-test	
Brawner 2022 [61]		Patients were attending CR for CABG, MI, PCI, valve replacement, heart failure	*n* = 4455 (2829 male, 1626 female)	Aerobic	≤ 18 weeks	9–36 visits 1–3 days/week	11	ANOVA	
Brochu 2000 [29]		Patients with AMI, CABG, unstable angina, and CHF	*n* = 82 (59 men, 23 women)	Aerobic and Strength	12 Weeks	CR completion: 100%	12	ANOVA	
Calvo-Lopez 2023 [62]		Patients with acute MI in last 3 months	*n* = 50 (42 men, 8 women)	Home-based (Aerobic and Strength)	8 Weeks	CR completion: 100% Adherence was 82.9% men, 95.6% women	11	t-test between deltas	
Caminiti 2022 [104]		Patients with CAD	*n* = 55 (35 men, 20women)	Aerobic and Strength	12 Weeks	CR completion: 95% Adherence was 89.4% men and 86.1% women	12	ANOVA	
Candelaria 2020 [66]		Patients were eligible if they were attending CR for CAD, arrhythmia, heart failure and implantable devices	*n* = 849 (604 men, 245 women)	Aerobic and Strength	6–12 Weeks	CR completion: 71% 426/604 men, 74% 181/245 women	10	t-test	
Cannistra 1992 [56]		Patients with heart disease: MI +- revascularization, CABG, coronary angioplasty, or angina pectoris	*n* = 225 (174 men, 51 women)	Aerobic	12 Weeks	CR completion: 63% 110/174 men, 51% 26/51 women. Adherence was 85 ± 11% for women and 89 ± 13% for men	10	t-test	
Casey 2009 [87]		Patients were CAD outpatients and as well as diagnosed with stable angina, MI, PCI, CABG, cardiomyopathy, or CHF	*n* = 637 (459 men, 178 women)	Not described	13 Weeks	CR completion: 92% 422/459 men completed study, 88% 157/178 women completed study	11	t-test between deltas	
Caulin-Glaser 2005 [97]		Patients with CAD who participated in a CR program	*n* = 172 (134 men, 38 women)	Not described	12 Weeks	CR completion: 100% (must have completed > 7 weeks)	11	t-test between deltas	
Caulin-Glaser 2007 [42]		Patients with CABG, PTCA, MI/CAD, or Valve disease	*n* = 348 (248 men, 100 women)	Not described	12 Weeks	CR completion: 90% 224/248 men, 71% 71/100 women	12	t-test between deltas	
Chai 1999 [43]		Patients with recent MI, CABG or angioplasty or stable angina pectoris	*n* = 113 (89 men, 24 women)	Aerobic and Strength	12 Weeks	CR completion: 100%	9	t-test	
Dehgani 2021 [79]		Patients with MI and HF	*n* = 40 (20 men, 20 women)	Home-based (Aerobic)	8 Weeks	CR completion: 100%	11	t-test	
Deljanin-Ilic 2019 [105]		Patients with MI, CABG, PCI	*n* = 684 (506 men, 178 women)	Aerobic	3 weeks	CR completion: 100%	11	t-test	
El Missiri 2020 [72]		Patients with CAD and underwent PCI 3 months prior to their enrollment in the study	*n* = 60 (30 men, 30 women)	Aerobic	12 Weeks	CR completion: 100% Adherence rate for men was 79.6% and 65.4% for women	12	t-test	
Fiorina 2007 [69]		Patients had either CABG, valve replacement, or AMI	*n* = 1622 (1123 men, 499 women)	Aerobic	15 ± 3 Days	CR completion: 100%	12	t-test	
Freene 2018 [70]		Patients with stable CAD with/ without a revascularization procedure	*n* = 72 (57 men, 15 women)	Not described	6 Weeks	CR completion: 90%	11	t-test between deltas	
Gee 2014 [44]		Patients with stable angina, MI, PCI, or CABG	*n* = 1104 (758 men, 346 women)	Not described	12 Weeks	CR completion: 73% 554/758 men, 66% 227/346 women. Adherence rate for men was 73.1% and was 65.6% for women	12	t-test	
Ghashghaei 2012 [45]		Patients with CAD including having history of one or more of the following: MI, CABG, PCI, and chronic stable angina	*n* = 156 (men = 72, women *n* = 84)	Aerobic and Strength	8 Weeks	CR completion: 100%	11	t-test between deltas	
Goldhammer 2007 [71]		Patients with CAD, having a history of MI, PCI, and/or CABG 21 to 45 days prior to inclusion to study	*n* = 37 (23 men, 14 women)	Aerobic	12 Weeks	CR completion: 100%	12	ANOVA	
Gupta 2007 [68]		Patients with CAD and had either diabetes, dyslipidemia, hypertension or were obese	*n* = 533 (373 men, 160 women)	Aerobic	8–12 Weeks	CR completion: 100%	11	t-test	
Heald 2021 [26]		Patients with CAD, spontaneous coronary artery dissection, AF, adult congenital heart disease, cardiomyopathy, PCI, CABG, valve intervention, implantable rhythm device, aneurysm repair, ablation, and those at risk for developing CAD or cardiovascular disease	*n* = 997 (454 men, 543 women)	Aerobic and strength	26 weeks	CR completion: 62% men, 59% women	11	t-test and ANOVA	
Jafri 2023 [92]		Patients with NSTEMI, STEMI, PCI, CABG, angina, heart transplant, valve repair/replacement, or HF	*n* = 15,613 (8825 men, 6788 women)	Aerobic and strength	9 Weeks	CR completion: 65.9% men, 63.3% women	11	t-test	
Keating 2013 [106]		Patients with CAD and had not had a MI or been hospitalized within the past 3 months. Patients also had a BMI > 27 kg/m 2, and waist circumference > 102 cm (men) or > 88 cm (women)	*n* = 46 (35 men, 11 women)	Aerobic; Home based	16 Week exercise + 4 Week weight control and exercise	CR completion: 100%	9	ANOVA	
Keteyian 2003 [17]		Patients with HF caused by left ventricular systolic dysfunction (New York Heart Association class II or III, a resting ejection fraction of < 35%)	*n* = 15 (10 men, 5 women)	Aerobic	14–24 weeks	CR completion: 100%	11	t-test between deltas	
Kim 2019 [37]		Patients who underwent PCI after MI or unstable angina visited the CR center at 4 weeks after being discharged from the hospital	*n* = 586 (451 men, 135 women)	Aerobic	36 Weeks	CR completion: 25% 114/451 men, 21% 30/135 women. Adherence rates between men and women were similar	11	t-test	
Kitagaki 2022 [41]		Patients with MI	*n* = 156 (128 men, 28 women)	Aerobic and Strength	12 Weeks	CR completion: 100% Men attended 10/36 classes and women attended 14/36 classes.	11	t-test and ANOVA	
Kligfield 2003 [46]		Patients who were referred for a 12-week program of CR because of stable angina or after they experienced MI, CABG, or PCI	*n* = 81 (58 men, 23 women)	Aerobic	12 Weeks	CR completion: 100%	11	Not described	
Kodis 2001 [18]		Patients with CABG	*n* = 1042- 713 in-person CR (612 men; 101 women) 329 home-based CR (296 men; 33 women)	Aerobic and Strength or Home-Based	24 Weeks	In person CR completion: 84% 516/612 men, 82% 83/101 women; Home-based CR completion: 97% 288/296 men, 91% 30/33 women	12	ANOVA	
Korzeniowska-Kubacka 2015 [57]		Patients with stable angina pectoris (CCS class I or II), sinus rhythm, preserved left ventricular function (ejection fraction > 50%), no evidence of right ventricular dysfunction on echocardiography, and those who were qualified for phase II of a comprehensive, post-MI, CR program	*n* = 87 (57 men, 30 women)	1/2 Aerobic, 1/2 Home Based	8 Weeks	CR completion: 100%	12	t-test	
Korzeniowska-Kubacka 2017 [81]		Patients who experienced MI within last 3 months, sinus rhythm, preserved left ventricular function (ejection fraction > 50%), and referred to CR	*n* = 62 (32 men, 30 women)	Aerobic	8 Weeks	CR completion: 100%	12	t-test and ANOVA	
Lavie 1995 [47]		Patients with CAD	*n* = 458 (375 men, 83 women)	Aerobic	12 Weeks	CR completion: 100%	12	t-test and ANOVA	
Lee 2018 [19]		Patients with CAD	*n* = 1544 (1359 men, 185 women)	Aerobic + Strength Vs Aerobic Interval Training	24 Weeks	CR completion: 73%	11	t-test	
MacMillan [30] 2006		Patients who suffered from MI, CABG, PCI, stable angina, revascularization, HF or valve replacement	*n* = 100 (69 men, 31 women)	Aerobic	12 Weeks	CR completion: 100%	11	t-test	
Maugeri 1998 [107]		Patients with HF (NYHA class III)	*n* = 134 (126 men, 8 women)	Aerobic	6–16 Weeks	CR completion: 94%	10	t-test and ANOVA	
McConnell 1997 [58]		Patients had to have a diagnosis of MI or coronary artery revascularization bypass surgery	*n* = 581 (456 men, 125 women)	Aerobic	12 Weeks	76% adherence for body composition, 36% for lipid measures, 20% for VO_2_	12	ANOVA	
Mertens 1996 [40]		Patients with chronic AF, and/or had a history of valvular disease, or CAD	*n* = 20 (13 men, 7 women)	Aerobic	52 Weeks	CR completion: 100%	9	Not described	
Mittag 2006 [90]		Patients suffered with MI, CABG, and PCI	*n* = 343 (281 men, 62 women)	Home-based	52 Weeks	CR completion: 87%	12	t-test	
Mroszczyk-McDonald 2007 [20]		Patients who suffered from CABG, MI, PCI, HF, and/or diagnosed with stable angina	*n* = 1960 (men = 1479, women = 481)	Aerobic and Strength	12 Weeks	CR completion: 34% 505/1479 men, 33% 161/481 women	10	t-test and ANOVA	
Nguyen 2021 [21]		Patients diagnosed with CAD	*n* = 63 (40 men, 23 women)	Aerobic and Strength	24 Weeks	CR completion: 100%	12	ANOVA	
Ocallaghan 1984 [85]		Patients who had MI or PCI	*n* = 264 (227 men, 37 women)	Aerobic	8 Weeks	CR completion: 92% of men, 81% of women. Men had an attendance rate of 87 ± 14% and women 77 ± 16%.	11	t-test	
O’Farrell 2000 [75]		Patients with either angina, MI, PTCA, CABG, CHF, cardiomyopathy, valvular disease, or heart transplantation	*n* = 387 (317 men, 70 women)	Aerobic	12 Weeks	CR completion: 100%. Men attended 83%, while women attended 76%.	12	t-test	
Pabisiak 2013 [108]		Patients who suffered a MI	*n* = 61 (34 men, 27 women)	Aerobic and Strength	8 Weeks	CR completion: 100%	11	t-test	
Pina 2014 [35]		Patients with HF, patients with left ventricular dysfunction (ejection fraction < 35%) and NYHA class 2 to 4	*n* = 2331 (1670 men, 661 women)	Aerobic	12 Weeks	CR completion: 81% of men and 79% of women. Adherence was 45% for men and 37% for women	12	t-test	
Pischke 2006 [59]		Patients had a diagnosis of CAD, and a history of CABG, PTCA, angina or MI	*n* = 343 (286 men, 57 women)	Aerobic	12 Weeks	CR completion: 95% of men completed the study and 95% of women.	12	ANOVA	
Proenca 2023 [93]		Patients had ACS	*n* = 881 (739 men, 142 women)	Not described	8–16 Weeks	CR completion: 100%	9	Unknown	
Quindry 2022 [94]		Patients had MI, CABG, PCI, Angina, HF, valve replacement, or have a heart transplant	*n* = 31,885 (22,602 men, 9283 women)	Not described	12–36 sessions (length unclear)	CR completion: 100%	12	ANOVA and Mann Whitney U test	
Rejeski 2002 [60]		Patients with a history of MI, PTCA, chronic stable angina, New York Heart Association Class I or Class II CHF, cardiovascular surgery (coronary artery or valvular heart disease) in the past 6 months	*n* = 147 (76 men, 71 women)	Aerobic and Strength	12 Weeks	CR completion: 88%	12	ANOVA	
Rengo 2020 [22]		Patients with a history of CABG, MI, PCI, CHF, stable angina, arrhythmia, and valve surgery	*n* = 3925(2985 men, 940 women)	Aerobic (some CR had HIIT)	12–16 Weeks	CR completion: 46% of men, 43% of women	12	t-test and ANOVA	
Sadeghi 2012 [48]		Patients with a history of MI, CABG, PCI, and chronic stable angina	*n* = 585 (men = 464, women = 121)	Aerobic and Strength	8 Weeks	CR completion: 100%	9	t-test between deltas	
Saeidi 2013 [49]		Patients with a history of MI, PTCA, CABG, and CAD	*n* = 100 (69 men, 31 women)	Aerobic	8 Weeks	CR completion: 100%	9	t-test	
Safdar 2022 [6]		Patients with CABG	*n* = 420 (344 men, 76 women)	Aerobic and Strength	12 weeks	CR completion: 32% of men, 23% of women.	11	t-test	
Sarrafzadegan 2008 [50]		Patients had a history of MI, CABG, PCI or CAD	*n* = 547 (400 men, 147 women)	Aerobic	8 Weeks	CR completion: 45%	12	t-test and ANOVA	
Savage 2004 [23]		Patients had a history of MI, CABG, CHF, unstable angina or CAD	*n* = 340 (263 men, 77 women)	Aerobic + Strength	12 Weeks	CR completion: 100%	12	t-test and ANOVA	
Sheikhian 2018 [98]		Patients with CAD, at least one coronary artery stenosis, an ejection fraction > 40% without evidence of ischemia or CHF symptoms	*n* = 30 (15 men, 15 women)	Aerobic	8 Weeks	CR completion: 100%	12	t-test and ANOVA	
Shultz 2010 [25]		Patients who had a history of CABG, MI, PTCA and/or valve surgery	*n* = 109 (60 men, 49 women)	Aerobic	12 Weeks	CR completion: 100%	11	t-test	
Socha 2017 [95]		Patients with CAD following CABG	*n* = 65 (44 men, 21 women)	Aerobic	3 Weeks	CR completion: 100%	11	Other (Wilcoxon)	
Soleimani2009a [84]		Patients with CAD who had previously undergone elective PTCA	*n* = 440 (339 men, 101 women)	Aerobic	8 Weeks	CR completion: 59% of men, 85% of women. 19.3% of patients attended 24 or more sessions	10	t-test	
Soleimani 2009b [76]		Patients with CAD	*n* = 216 (168 men, 48 women) -nondiabetics	Aerobic	8 Weeks	CR completion: 100% of men, 81% of women	11	t-test	
Stojanovic 2023 [82]		Patients with MI, PCI, or myocardial revascularization	*n* = 1603 (1231 men, 372 women)	Aerobic	3 Weeks	CR completion: 100%	11	t-test	
Swank 2010 [77]		Patients with CHF, ejection fraction < 35%, with systolic dysfunction	*n* = 42 (27 men, 15 women)	Aerobic and Strength	14 Weeks	CR completion: 100%	12	ANOVA	
Szmigielska 2022 [52]		Patients with CAD who had CABG, PCI, or ACS	*n* = 286 (180 men, 106 women)	Aerobic	8 Weeks	CR completion: 100%	11	t-test and t-test between deltas	
Temfemo 2011 [31]		Patients who had a history of CABG, artery angioplasty, MI, or valve replacement	*n* = 188 (112 men, 76 women)	Aerobic	8 Weeks	CR completion: 100%	12	ANOVA	
Terada 2019a [89]		Patients with a history of CAD, arrhythmia, valvular disease, AF, and/or CHF	*n* = 120 (60 men, 60 women)	Aerobic (Either AIT or MICE)	8 Weeks	CR completion: 100%	12	t-test and ANCOVA	
Terada 2019b [78]		Patients diagnosed with angina, CABG, PCI, CAD, CHF, cardiomyopathy, heart murmur, carotid disease, or transient ischemic attack	*n* = 591 (436 men, 155 women)	Aerobic (On site, home based, or brief)	4–12 weeks	CR completion: 100% Adherence: 85% men and 83% women (on-site)	12	t-test and ANCOVA	
Thorin-Trescases 2016 [24]		Patients diagnosed with ACS	*n* = 40 (30 men, 10 women)	Aerobic	12 Weeks	CR completion: 100%. Adherence rate for men was 98.9 ± 3.0% and for women 97.0 ± 3.4%	12	ANCOVA and Other (Wilcoxon)	
Trachsel 2020 [32]		Patients with CAD	*n* = 83 (64 men, 19 women)	Aerobic and Strength	12-14Weeks	CR completion: 88% of men, 86% of women completed	12	ANOVA	
Turk-Adawi 2016 [91]		Patients with ACS, revascularization, stable HF, and heart valve repair or replacement	*n* = 12,976 (8,836 men, 4,140 women)	Not described	12 Weeks	CR completion: 71% of men, 65% of women	12	ANOVA	
Tyni-Lenne 1998 [39]		Patients with CHF, ejection fraction < 40%, New York Heart Association (NYHA) functional class II and class III	*n* = 24 (12 men, 12 women)	Knee extensor exercise	8 Weeks	CR completion: 100% Adherence to program ranged from 86–100% with no sex-related differences.	12	t-test and ANOVA	
Verrill 2003 [67]		Patients with CAD, and/or angina, valvular disease or replacement, CHF or MI	*n* = 630 (424 men and 206 women)	Aerobic and Strength	10–12 Weeks	CR completion: 100%	12	ANOVA	
Vidal-Almela 2022 [36]		Patients with CAD, arrhythmias, valvular disease, angina, stroke, HF, spontaneous coronary artery dissection, PCI, CABG, valve replacement, PCI + CABG, catheter ablation	*n* = 140 (100 men, 40 women)	Aerobic (HIIT)	10 weeks	CR completion: 100% Adherence 78% men, 77% women	11	ANCOVA	
Vilela 2020 [33]		Patients who suffered from AMI	*n* = 379 (307 men, 72 women)	Aerobic and Strength	8 Weeks	CR completion: 100%. Adherence: Patients completed a median of 22 sessions (16–25 sessions)	12	t-test	
Wagner 2018 [34]		Patients with AF	*n* = 210 (151 men, 59 women)	Aerobic and Strength	12 Weeks	CR completion: 82% of men, 88% of women. Adherence to the training sessions was higher in men (47%) than in women (40%)	11	t-test	
Wahlstrom 2023 [80]		Patients with AF	*n* = 64 (28 men, 36 women)	Yoga	12 Weeks	CR completion: 100%	11	t-test	
Warner 1995 [88]		Patients were referred after MI, CABG, percutaneous balloon angioplasty, cardiomyopathy, and/or stable angina	*n* = 719 (553 men 166 women)	Aerobic	260 Weeks	CR completion: 12% 69/553 men, 8% 14/166 women. Adherence was 70% for men and 68% for women	12	t-test and ANCOVA	
Weinberger 2014 [86]		Patients with PTCA, CABG, MI, valve replacement, CAD, CHF, angina or cardiomyopathy	*n* = 1138 (843 men, 295 women)	Not described	≥ 7weeks; average 11 weeks	CR completion: 49% Adherence was 11.5 weeks for men and 11.4 weeks for women	12	Other (Logistic regression)	
Willenheimer 1998 [38]		Patients with HF and ejection fraction < 45%	*n* = 33 (27 men, 6 women)	Aerobic	16 Weeks	CR completion: 91% Adherence for men was 78% and for 67% women	12	t-test	
Wise 2012 [51]		Patients with CHF (NYHA level I-III) also diagnosed with CAD, cardiomyopathy, valvular disease, and or/tachycardia	*n* = 232 (172 men, 60 women)	Aerobic and Strength	12 Weeks + median of 4 extra weeks	CR completion: 100% All patients attended at least 40% of sessions.	11	Other (Wilcoxon)	
Zahedi 2022 [74]		Patients with MI and PCI	*n* = 30 (23 men, 7 women)	Aerobic	5 Weeks	CR completion: 100%	9	Other (Wilcoxon)	
Zahedi 2023 [73]		Patients with CABG	*n* = 100 (67 men, 33 women)	Aerobic	4 weeks	CR completion: 100%	11	Other (Wilcoxon)	

ACS; acute coronary syndrome, AF; atrial fibrillation, AMI; acute myocardial infarction, BMI; body mass index, CABG; coronary artery bypass grafting, CAD; coronary artery disease, CHF; chronic heart failure, CR; cardiac rehabilitation, HF; heart failure, HIIT; high intensity interval training, MI; myocardial infarction, NYHA; New York heart association PCI; percutaneous coronary intervention, PTCA; percutaneous transluminal coronary angioplasty

**Table 2:** Sex differences or similarities in the physiological responses to CR completion

Physiological response	Greater improvement in men	Only men improved	Greater improvement in women	Only women improved	Both men and women improve	No improvement in either sex
**Fitness**						
Maximal exercise VO_2_ (ml/kg/min)	Heald 2021, Keteyian 2003, Kodis 2001, Lee 2018, Mroszczyk-McDonald 2007, Nguyen 2021, Rengo 2020, Savage 2004, Shultz 2010, Thorin-Trescases 2016 **(10 studies)**	Kim 2019, Willenheimer 1998 **(2 studies)**		Tyni-Lenne 1998 **(1 study)**	Ades 1992, Antunes-Correa 2010, Brochu 2000, MacMillan 2006, Piepoli 1998, Pina 2014, Temfemo 2011, Trachsel 2020, Vidal-Almela 2022, Vilela 2020, Wagner 2018 **(10 studies)**	
Percent-predicted peak VO_2_ (%)	Mertens 1996 **(1 study)**	Kitagaki 2022 **(1 study)**			Trachsel 2020, Vilela 2020 **(2 studies)**	
RER ratio			Rengo 2020 **(1 study)**		Trachsel 2020, Vilela 2020 **(2 studies)**	Ades 1992 **(1 study)**
Functional Capacity (METS)	Caulin-Glaser 2007, Chai 1999, Gee 2014, Ghashghaei 2012, Heald 2021, Kligfield 2003, Kodis 2001, Lavie 1995, Sadeghi 2012, Saeidi 2013, Sarrafzadegan 2008, Szmigielska 2022, Wise 2012 **(13 studies)**	Safdar 2022, Willenheimer 1998 **(2 studies)**			Anjo 2014, Balady 1996, Branco 2016, Cannistra 1992, Korzeniowska-Kubacka 2015, MacMillan 2006, Pischke 2006, Rejeski 2002, Brawner 2022, Calvo-Lopez 2023 **(10 studies)**	
6MWD (m)	Araya-Ramirez 2021, Bellet 2015 Candelaria 2020, Gupta 2007, Tyni-Lenne 1998, Verrill 2003 **(6 studies)**		Wagner 2018, Wise 2012 **(2 studies)**		Fiorina 2007, Freene 2018, Kim 2019 Rejeski 2002 **(4 studies)**	
Peak workload/LBM (W/kg)	Kodis 2001 **(1 study)**				Szmigielska 2022, Trachsel 2020, Tyni-Lenne 1998 **(3 studies)**	
HR at rest (bpm)	ElMissiri 2020, O’Farrell 2000, Saeidi 2013 **(3 studies)**	Anjo 2014, Szmigielska 2022 **(2 studies)**	Korzeniowska-Kubacka 2017 **(1 study)**		Cheragi 2021, Goldhammer 2007, Pischke 2006,, Soleimani- Nejatian 2009, Swank 2010, Temfemo 2011, Terada 2019 **(7 studies)**	Ades 1992, Antunes-Correa 2010, Araya-Ramirez 2021, Korzeniowska-Kubacka 2015, Lavie 1995, MacMillan 2006, Sarrafzadegan 2008, Tyni-Lenne 1998, Wahlstrom 2023 **(9 studies)**
Maximal HR (bpm)				Keteyian 2003, Lavie 1995 **(2 studies)**	Cheragi 2021, Goldhammer 2007, Korzeniowska-Kubacka 2015, MacMillan 2006, Soleimani-Netajian 2009, Temfemo 2011 **(6 studies)**	Ades 1992 **(1 study)**
Difference in HRR (bpm)	Kligfield 2003 **(1 study)**				Anjo 2014, Araya-Ramirez 2021, MacMillan 2006, Soleimani-Nejatian 2009 **(4 studies)**	
Resting rate pressure product (beats/min x mmHg)		Stojanovic 2023 **(1 study)**			Szmigielska 2022 **(1 study)**	Ades 1992 **(1 study)**
Peak rate pressure product (beats/min x mm Hg)	Cannistra 1992 **(1 study)**	Szmigielska 2022 **(1 study)**			Cheragi 2021, Stojanovic 2023, Swank 2010 **(3 studies)**	Ades 1992, Lavie 1995 **(2 studies)**
Exercise time (s)			Wagner 2018 **(1 study)**	Safdar 2022 **(1 study)**	Ades 1992, Balady 1996, Cannistra 1992, Goldhammer 2007, Korzeniowska-Kubacka 2015, Ocallaghan 1984, Stojanovic 2023, Vilela 2020 **(8 studies)**	
Absolute energy expenditure (kcal/wk)					O’Farrell 2000, Schultz 2010, Soleimani 2009 **(3 studies)**	
Grip strength (kg)	Mroszczyk-McDonald 2007, Rengo 2020 **(2 studies)**	Kim 2019, Safdar 2022 **(2 studies)**				
Sit to stand (number)	Wagner 2018 **(1 study)**	Kim 2019 **(1 study)**			Calvo-Lopez 2023 **(1 study)**	
**Metabolic Measures**						
HDL (mg/dl)	Weinberger 2014 **(1 study)**	Gupta 2007, Sadeghi 2012 **(2 studies)**	Casey 2009, Heald 2021, Savage 2004, Terada 2019 **(4 studies)**	Warner 1995 **(1 study)**	Anjo 2014, Brochu 2000, Caulin-Glaser 2007, Ghashghaei 2012, Kim 2019, Kitagaki 2022, Lavie 1995, Mittag 2006, O’Farrell 2000, Pischke 2006, Sarrafzadegan 2008, Shultz 2010, Vidal-Almela 2022 **(13 studies)**	Goldhammer 2007, Szmigielska 2022, Thorin-Trescases 2016, Turk-Adawi 2016 **(4 studies)**
Total cholesterol: HDL ratio					O’Farrell 2000, Vidal-Almela 2022, Warner 1995 **(3 studies)**	Brochu 2000, Thorin-Trescases 2016 **(2 studies)**
Total Cholesterol (mg/dl)	Casey 2009, ElMissiri 2020, Ghashghaei 2012, Jafri 2023 **(4 studies)**				Anjo 2014, Brochu 2000, Gupta 2007, Kim 2019, Lavie 1995, McConnell 1997, Mittag 2006, O’Farrell 2000, Pischke 2006, Sadeghi 2012, Sarrafzadegan 2008, Savage 2004, Turk-Adawi 2016, Warner 1995 **(14 studies)**	Goldhammer 2007, Heald 2021, Szmigielska 2022, Thorin-Trescases 2016, Vidal-Almela 2022 **(5 studies)**
LDL (mg/dl)	Jafri 2023, Proenca 2023 **(2 studies)**				Anjo 2014, Casey 2009, Caulin-Glaser 2007, ElMissiri 2020, Ghashghaei 2012, Goldhammer 2007, Gupta 2007, Kitagaki 2022, O’Farrell 2000, Pischke 2006, Sadeghi 2012, Sarrafzadegan 2008, Savage 2004,Terada 2019, Thorin-Trescases 2016, Turk-Adawi 2016, Vidal-Almela 2022, Warner 1995 **(18 studies)**	Brochu 2000, Heald 2021, Kim 2019, Shultz 2010, Szmigielska 2022 **(5 studies)**
LDL-C/HDL-C					Ghashghaei 2012, Lavie 1995, O’Farrell 2000 **(3 studies)**	
TGs (mg/dl)	ElMissiri 2020, Jafri 2023, Turk-Adawi 2016 **(3 studies)**	Szmigielska 2022 **(1 study)**			Anjo 2014, Casey 2009, Lavie 1995, McConnell 1997, Pischke 2006, Sadeghi 2012, Sarrafzadegan 2008 Savage 2004, Shultz 2010, Terada 2019, Turk-Adawi 2016, Vidal-Almela 2022, Warner 1995 **(13 studies)**	Brochu 2000, Caulin-Glaser 2007, Goldhammer 2007, Heald 2021, Kim 2001, Kitagaki 2022, O’Farrell 2000, Thorin-Trescases 2016, **(8 studies)**
SBP (mmHg)	Mittag 2006 **(1 study)**		O’Farrell 2000, Turk-Adawi 2016 **(2 studies)**	Wahlstrom 2023 **(1 study)**	Araya-Ramirez 2021, Casey 2009, Cheragi 2021, Jafri 2023, Pischke 2006, Quindry 2022, Sarrafzadegan 2008, Shultz 2010, Swank 2010, Szmigielska 2022, Terada 2019, Thorin-Trescases 2016 **(12 studies)**	Ades 1992, Calvo-Lopez 2023, Heald 2021, Kim 2019, Korzeniowska-Kubacka 2015, Lavie 1995, Terada 2019, Trachsel 2020, Vidal-Almela 2022 **(9 studies)**
DBP (mmHg)			ElMissiri 2020, Trachsel 2020 **(2 studies)**		Araya-Ramirez 2021, Casey 2009, Cheragi 2021, Pischke 2006, Quindry 2022, Sarrafzadegan 2008, Szmigielska 2022, Terada 2019, Thorin-Trescases 2016, Wahlstrom 2023 **(10 studies)**	Calvo-Lopez 2023, Heald 2021, Kim 2019, Korzeniowska-Kubacka 2015, O’Farrell 2000, Shultz 2010, Swank 2010, Terada 2019, Turk-Adawi 2016, Vidal-Almela 2022 **(10 studies)**
Glucose (mg/dl)		Ghashghaei 2012, Szmigielska 2022 **(2 studies)**	Anjo 2014, **(1 study)**		Sarrafzadegan 2008, Shultz 2010, Terada 2019, Vidal-Almela 2022 **(4 studies)**	Brochu 2000, Goldhammer 2007, O’Farrell 2000, Thorin-Trescases 2016 **(4 studies)**
Changes in Glycated hemoglobin A1C (%)	Jafri 2023 **(1 study)**				Anjo 2014, Terada 2019, Turk-Adawi 2016 **(3 studies)**	Kitagaki 2022, Vidal-Almela 2022 **(2 studies)**
**Body Composition**						
Weight (kg)	Ghashghaei 2012, Terada-Chirico 2019 **(2 studies)**				Casey 2009, McConnell 1997, Mroszczyk-McDonald 2007, Pischke 2006, Rengo 2020, Savage 2004, Socha 2017, Temfemo 2011, Terada 2019, Thorin-Trescases 2016 **(13 studies)**	Araya-Ramirez 2021, Brochu 2000, Jafri 2023, Shultz 2010, Vidal-Almela 2022, **(5 studies)**
Waist Circumference (cm)	Brochu 2000 **(1 study)**		Braga 2021 **(1 study)**		Casey 2009, ElMissiri 2020, Heald 2021, Mroszczyk-McDonald 2007, Rengo 2020, Sarrafzadegan 2008, Savage 2004, Shultz 2010, Terada 2019, Thorin-Trescases 2016, Vidal-Almela 2022 **(11 studies)**	Kim 2019, Szmigielska 2022, Turk-Adawi 2016 **(3 studies)**
BMI (kg/m^2^)		Heald 2021, Szmigielska 2022 (**2 studies**)	Sarrafzadegan 2008 **(1 study)**		Anjo 2014, Brochu 2000, Casey 2009, Caulin-Glaser 2007, ElMissiri 2020, Gupta 2007, Jafri 2023, Lavie 1995, Mroszczyk-McDonald 2007, Rengo 2020, Savage 2004, Socha 2017, Terada 2019, Thorin-Trescases 2016 **(14 studies)**	Araya-Ramirez 2021, O’Farrell 2000, Shultz 2010, Turk-Adawi 2016 **(4 studies)**
Body fat (%)	Brochu 2000 **(1 study)**				Lavie 1995, McConnell 1997, Pischke 2006, Socha 2017, Thorin-Trescases 2016 **(5 studies)**	Kim 2019 **(1 study)**
Total fat mass (%)	Brochu 2000 **(1 study)**				Thorin-Trescases 2016 **(1 study)**	Safdar 2022 **(1 study)**
**Cardiac Measures**						
LVEF (%)		Zahedi 2023 **(1 study)**			ElMissiri 2020, Saeidi 2013, Sarrafzadegan 2008, Szmigielska 2022, Zahedi 2023 **(5 studies)**	Antunes-Correa 2010, Goldhammer 2007, Trachsel 2020 **(3 studies)**
**Protein Measures**						
CRP (mg/L)		Thorin-Trescases 2016 **(1 study)**			Caulin-Glaser 2005, Goldhammer 2007, Sheikhian 2018 **(3 studies)**	

BMI; body mass index, CRP; c-reactive protein, DBP; diastolic blood pressure, HDL; high density lipoprotein, HR; heart rate, HRR: heart rate recovery, LBM; lean body mass, LDL; low density lipoprotein, LVEF; left ventricular ejection fraction, METS; metabolic equivalents, RER; respiratory exchange ratio, SBP; systolic blood pressure, TGs; triglycerides, 6MWD; 6-minute walk distance

The study design was characterized as one of the following: cohort study, retrospective cohort, retrospective cross-sectional, or randomized/non-randomized controlled trial. Population description presented the type of HD in the sample, with most studies investigating CAD populations. The total number of participants included the number of men and women in each population where available. Furthermore, the type of CR stated the nature of the exercise, including aerobic-only, aerobic and strength, home-based, or were not described. Typically, the articles that did not describe the exact exercise intervention were chart reviews from multiple CR programs. The length of CR specified the length of the intervention in weeks, ranging from 2 to 260 weeks. The median number of weeks of CR intervention was 12, with every intervention incorporating at least aerobic exercise (unless the intervention was unknown). The most prevalent type of CR was aerobic-only, followed by aerobic and strength exercise. The study populations generally had higher enrollment and participation rates in men compared to women, whereas adherence appeared similar between sexes.

The average QA for all articles was 11.2 ± 0.9 out of 12; 41 articles scored 12/12, 33 articles scored 11/12, 7 scored 10/12, and 7 scored 9/12. The CR completion and adherence rates were calculated and separated based on sex where available. Lastly, the type of statistics included repeated measures ANOVAs between sexes over time (i.e. “ANOVAs”; this category also included generalized linear models and generalized estimated equations analysis), t-tests over time within only one sex (i.e. “t-test”), t-tests between sexes on the change from pre- to post-CR (i.e. “t-test between deltas”), other types such as Wilcoxon signed rank test, or the statistical methods were not described. While ANCOVAs using sex only as a covariate were excluded, we retained studies that analyzed disaggregated sex data as the main variable but had other factors, such as age as a covariate.

Table 2 provides a tabular quantification of the number of articles which described each physiological variable indicating (1) a greater improvement in men, (2) only men improved, (3) greater improvement in women, (4) only women improved, (5) men and women both improved, or (6) neither men nor women improved. The variables were divided into 5 physiological groups, including fitness, metabolic measures, body composition, cardiac measures, and C-reactive protein for appropriate comparisons.

## Discussion

### Oxygen consumption at maximal exercise

Many studies have indicated a greater improvement in maximal oxygen consumption (VO_2_ max (ml/kg/min)) after CR in men compared to women [17–26], while other studies argued no sex difference in this physiological response [27–36]. Although both sets of articles have different outcomes regarding maximal oxygen consumption, the types of exercise were generally similar, including either aerobic and/or strength. Thus, the type of exercise is not a strong enough factor to predict whether men and women improve similarly. VO_2_max was found to only improve in men and not women in 2 additional studies [37, 38]. Kim & So utilized an extended CR program length of 36 weeks [37]. This was the longest intervention duration among articles that compared VO_2_max responses and could be a determining factor, as many articles stating no sex difference had interventions ranging only from 8 to 12 weeks [27, 29–35]. Secondly, in Willenheimer et al., only patients with heart failure (HF) were studied [38], whereas the studies concluding no sex difference looked at populations with various HDs. Thus, program length or type of HD could potentially play a role in the observation of sex differences. Interestingly, Tyni-Lenne et al. investigated patients with HF enrolled in an 8-week intervention of knee extensor exercise [39] and observed an improvement of VO_2_max only in women. This type of exercise is strictly experimental rather than clinical, thus it would not typically be used in isolation during a CR program. Improvements in VO_2_max from this exercise intervention could potentially be due to increased strength and therefore greater daily physical activity. When comparing %-predicted peak VO_2_, Mertens & Kavanagh found a greater improvement in men versus women after CR [40] and Kitagaki et al. found that only men improved after CR [41]; however, two studies by Trachsel et al., and Vilela et al., concluded that this percentage improves equally in both sexes [32, 33]. Mertens & Kavanagh (*n* = 7/20 women), Kitagaki et al. (*n* = 28/156 women), and Trachsel et al. (*n* = 19/83 women) all had small numbers of women in their investigation cohorts whereas Vilela et al. had *n* = 72/379. The discrepancies in this case could be due to limited sample sizes.

### Functional capacity

Thirteen studies concluded that men experience a greater improvement in functional capacity (metabolic equivalents (METS)) than women post-CR [18, 26, 38, 42–52]. Conversely, 10 other articles observed that functional capacity improved equally in men and women after CR [30, 53–62]. When comparing the groups of studies, those that refuted a sex difference had similar CR interventions with similar duration and completion rates compared to the studies that found a sex difference. Based on the parameters collected in the scope of this review, it is undetermined what may distinguish the articles which found a sex difference from the articles which found similar responses between the sexes in functional capacity post-CR. However, as it was not in the scope of the current review, we did not extract all the information that could influence functional capacity, such as exercise intensity. This is a limitation of the current review since greater exercise intensity is associated with improved cardiorespiratory fitness and strength [63].

### Six minute walk distance (6MWD)

Six studies found that men experienced a greater improvement in 6MWD after CR compared to women [39, 64–68]. Four other studies refuted this sex difference and reported similar improvements in 6MWD between men and women [37, 60, 69, 70]. Disease or intervention type could not predict whether there is a sex difference in 6MWD outcomes, and the two groups of articles also used similar statistics with similar adherence rates. On the contrary, 2 additional studies by Wagner et al. and Wise et al. have reported that women have a greater improvement than men in 6MWD after CR [34, 51]. Wagner et al. only investigated individuals with atrial fibrillation in a randomized controlled trial, and Wise et al. investigated patients with HF in a cohort study design. Both studies investigated an isolated HD type, which could be a determining factor as to why it was observed that women improved more than men. However, like the rest of the articles that stated no sex difference or that men improved more, these studies utilized similar training interventions with an average of 12 weeks CR, including aerobic and strength exercise.

### Cardiac measures

Eight studies comparing left ventricular ejection fraction in HD patients reported equal improvements between the sexes or no improvements at all after CR [28, 32, 49, 50, 52, 71–73] with a single study finding that men improved to a greater degree than women (only 5 weeks of CR) [74]. While three studies investigating HR at rest showed a greater improvement in men after CR [49, 72, 75], the majority of the articles investigating HR at rest showed no sex difference or no improvement in either sex [27, 28, 30, 31, 39, 47, 50, 57, 59, 64, 71, 76–80]. When comparing these opposing groups of papers, there were no striking differences in terms of disease type, CR duration, adherence rates and exercise type between articles. Two further studies by Anjo et al. and Szmigielska et al. found that only men with CAD improved resting HR after 8–12 weeks of CR [52, 53] and one study by Korzeniowska-Kubacka et al. found that there was a greater improvement in women after 8 weeks of CR [81]. Anjo et al. suggested that an impaired improvement in women could have occurred since their cohort of women were significantly older.

Keteyian et al. examined HF patients and Lavie et al. examined CAD patients, yet both found that only women experienced increased maximum HR during peak exercise after CR [17, 47]. However, most studies have found no sex difference or no improvement [27, 30, 31, 47, 57, 71, 76, 79]. Every article that discussed maximal HR had purely aerobic interventions; however, no sex differences were observed in studies that had populations of multiple HD types. We suggest that the increased post-maximal HR in women observed in Keteyian et al. and Lavie et al. could be due to greater maximal effort during the stress test since maximum HR is dictated primarily by age not training. Importantly, care needs to be taken when interpreting any changes in resting, maximal, or recovery HR in cardiac disease populations considering the potential use of pharmacological agents and/or the use of pacemakers.

Kligfield et al. looked at differences in heart rate variability in HD patients and concluded that there was a greater improvement in heart rate recovery (HRR) after CR in men compared to women [46]; however, four other studies found that both men and women improve equally. The major difference between Kligfield et al. and those that observed equal improvements could be due to their unspecified statistical methods. Indeed, the studies by Anjo et al., Araya-Ramirez et al., MacMillan et al., and Soleimani et al. utilized either ANOVAs or t-tests and did not support this conclusion; instead, they expressed that men and women experienced similar changes in HRR post-CR [30, 53, 64, 76]. The vast discrepancies in statistical analysis observed throughout this systematic review (without meta-analysis) were striking. We suggest that every research group investigating sex differences in CR programs should consult a biostatistician to determine appropriate analyses.

Rate pressure product (RPP) is the product of systolic blood pressure and heart rate and is an index of myocardial oxygen consumption. At rest, while Stojanovic et al. found that only men improved their RPP, both Szmigielska et al. and Ades et al. found that either both sexes improved equally or that there was no improvement at all [52, 82, 83]. During a peak exercise test, both Cannistra et al. and Szmigielska et al. found that men improved RPP [52, 56] whereas 5 other studies found either equal improvements or no improvement at all [47, 77, 79, 82, 83]. The bulk of evidence therefore suggests that men and women do not improve or improve RPP equally after completion of CR.

### Other fitness variables

Men and women responded similarly to CR in terms of absolute energy expenditure [25, 75, 84]. The remaining fitness variables included respiratory exchange ratio (RER), peak workload/lean body mass, exercise time, and grip strength. RER was found to improve more in women than men in Rengo et al., though studies by Ades et al., Trachsel et al., and Vilela et al. found equal improvements or no difference in improvement between men and women [22, 27, 32, 33]. Interestingly, in Rengo et al., the CR intervention included high-intensity interval training compared to the other studies that followed a typical continuous aerobic-only or aerobic and strength-based CR. Peak workload (normalized to lean body mass) during an exercise test was discussed and compared in studies by Trachsel et al., Kodis et al., Szmigielska et al. and Tyni-Lenne et al. [18, 32, 39, 52]. Most indicated no sex difference in peak workload improvement; however, Kodis et al. found a greater improvement in men compared to women. In the study by Kodis et al., only patients with CABG were studied, and they utilized a 24-week CR program, which is longer compared to the other 8–12 week interventions. Therefore, these findings suggest that sex differences may not become evident until a longer CR program is completed.

When comparing exercise time, Wagner et al. and Safdar et al. found that women with atrial fibrillation or CABG surgery, respectively, experienced a greater improvement than men, or only women improved [6, 34]. However, the bulk of studies found that men and women with multiple types of HD had similar improvements in exercise time and included a variety of aerobic, strength and home-based programs [33, 54, 56, 57, 71, 82, 85]. Thus, sex differences in the improvement in exercise time could potentially be dependent on the type of cardiac disease investigated. Importantly, grip strength was measured in four studies [6, 20, 22, 37], and men experienced a greater improvement than women in Mroszczyk-McDonald et al. and Rengo et al., while Kim & So and Safdar et al. found only an improvement in men. Therefore, the consensus reached is that men experienced a significantly greater improvement in handgrip after CR as compared to women.

### Cholesterol and triglycerides

Weinberger et al. concluded that HDL cholesterol levels improved more in men compared to women after CR [86]. However, the type of exercise prescribed during CR intervention was not specified in this study. Similarly, studies by Gupta et al. and Sadeghi et al. expressed that only men improved in HDL levels after CR [48, 68]. On the contrary, studies by Savage et al., Casey et al., Heald et al. and Terada et al. found that women had a greater improvement in HDL levels compared to men [23, 26, 78, 87]. Furthermore, a 5-year long cohort study by Warner et al. stated that only women improve their HDL levels after a CR intervention [88]. Women may have begun these latter studies with less favourable body composition, allowing them to manifest a greater HDL benefit [89]. Despite these conflicting observations on sex-related HDL improvement, the general consensus stated by most articles was that men and women appear to either improve equally in HDL levels after CR or see no improvement [24, 25, 29, 36, 37, 41, 42, 45, 47, 50, 52, 53, 59, 71, 75, 87, 90, 91].

Most of the articles in this review discussing LDL cholesterol, LDL: HDL, and total cholesterol: HDL concluded that no sex differences, or no improvements, in the response to CR were found [23–26, 29, 36, 37, 41, 42, 45, 47, 48, 50, 52, 53, 59, 68, 71, 72, 75, 78, 87, 88, 91]. However, interestingly 2 recent studies from Jafri et al. and Proenca et al. found that men improve LDL to a greater degree than women after CR [92, 93]. These were very large retrospective cohort studies of 15,613 and 881 cardiac patients, respectively. While the majority of studies found that no sex differences were evident in the improvement of total cholesterol after CR [23, 24, 26, 29, 36, 37, 47, 48, 50, 52, 53, 58, 59, 68, 71, 75, 88, 90, 91], El Missiri et al., Casey et al., Ghasghaei et al., and Jafri et al. all concluded that men experienced a greater improvement in total cholesterol levels compared to women [45, 72, 87, 92]. Adherence to the CR program could have played a role since the studies by Casey et al., El Missiri et al., and Jafri et al. found that women had lower completion/adherence rates compared to men; however, adherence was not discussed in the study by Ghashghaei et al. Participants with higher adherence are likely to experience greater benefit of CR participation. On the other hand, poor adherence or non-compliance with the prescribed regimen could impede progress.

Studies by El Missiri et al., Turk-Adawi et al., and Jafri et al. stated that there was a greater reduction in triglyceride levels in men compared to women after CR [72, 91, 92] and Szmigielska et al. similarly found that only men improved triglycerides after CR [52]. The remainder of the reviewed studies indicate equal improvements or no sex differences [23–26, 29, 36, 37, 42, 47, 48, 50, 53, 58, 59, 71, 75, 78, 87, 88, 91]. As each group of studies included a range of CR duration and a mix of aerobic and strength training it is unclear why a few studies found that women do not improve their triglycerides as much as men except perhaps for the very large sample sizes in Jafri et al. and Turk-Adawi et al. (> 10,000) This finding could further indicate that in order to observe sex differences in certain variables large populations must be studied.

### Systolic and diastolic blood pressure

Multiple references found that men and women improve equally (or do not improve) systolic blood pressure after CR [24–27, 32, 36, 37, 47, 50, 52, 57, 59, 62, 64, 77–79, 87, 89, 92, 94]. However, O’Farrell et al. and Turk-Adawi et al. observed a greater reduction in systolic blood pressure in women compared to men after 12 weeks of CR [75, 91], Wahlstrom et al. observed that only women reduced systolic blood pressure after 12 weeks of CR [80], and Mittag et al. observed a greater improvement in men after 52 weeks of CR [90]. Similarly, the majority of the research leaned towards no sex difference (or no improvement) in diastolic blood pressure response to CR [24, 26, 36, 37, 50, 52, 57, 59, 62, 64, 75, 77–80, 87, 89, 91, 94]. However, two studies by El Missiri et al., and Trachsel et al., stated that women improved their diastolic blood pressure more than men after CR [32, 72]. The outlier studies do not appear to be distinguished from the rest regarding CR duration, HD type, or other extracted variables; thus, it is difficult to identify the factor(s) driving these sex differences. However, the overwhelming majority of studies have indicated that after CR, blood pressure either improves equally between the sexes or no improvement in either sex is observed.

### Glucose & glycated hemoglobin

Most reviewed studies found that plasma glucose either did not improve after CR or that there were equal improvements in both sexes [24, 25, 29, 71, 75, 78]. However, a few studies did observe sex differences. Anjo et al. found that glucose measures improved more in women after completion of CR [53], yet women expressed worse glucose levels at baseline, thus eliciting a greater improvement after CR. Ghashghaei et al. and Szmigielska et al. observed that only men improved plasma glucose after CR [45, 52]. All of these outlier studies used t-test analyses and were perhaps statistically underpowered. Similar to the changes in plasma glucose, changes in glycated hemoglobin showed similar improvements (or no change) in men and women after CR [36, 41, 53, 89, 91], yet Jafri et al. recently found that men had a greater improvement in their large retrospective study [92].

### Body composition

Most studies have indicated that body composition measures (i.e. weight, waist circumference, BMI, body fat %, total fat mass) either improved equally between the sexes or did not improve at all after CR [6, 20, 22–25, 29, 31, 36, 37, 42, 47, 50, 52, 53, 58, 59, 64, 68, 72, 75, 87, 89, 91, 92, 95]. There were a few exceptions where Braga et al. and Sarrafzadegan et al. observed more improvement in waist circumference and BMI in women compared to men [50, 96], Heald et al. and Szmigielska et al. observed an improvement of BMI only in men [26, 52], and Terada et al., Ghashghaei et al. and Brochu et al. observed that there were greater improvements of body weight, body fat %, total fat mass, and waist circumference in men compared to women [29, 36, 78]. Brochu et al. hypothesized that their observations of greater improvements in men could stem from their observation that the women in their cohort were generally less fit than men [29]. Therefore, baseline fitness and physical activity levels could play a role in any observed sex differences.

### C-reactive protein

Thorin-Trescases et al. found that the levels of C-reactive protein (CRP; an inflammatory marker) were reduced in men after CR but not in women [24]. Three other studies, however, determined that no sex difference was found in CRP levels after CR [71, 97, 98]. Thorin-Trescases et al. investigated patients specifically with acute coronary syndrome, whereas the studies by Goldhammer et al., Sheikhian et al., and Caulin-Glaser et al. all investigated patients with CAD. Therefore, the sex differences observed by Thorin-Trescases et al. could be due to the type of HD investigated.

### Limitations

The current review has broadly described sex differences in the physiological responses to a wide range of CR programs. We did not limit the search with regards to physiological response, program length, program intensity, or cardiac disease type with the purpose of searching for trends that currently exist in the literature. A more targeted approach could have enhanced the precision of the findings, enabling a more comprehensive understanding of select research topics. However, limiting our search parameters to programs at least 12 weeks long would have reduced the number of studies to 46/88; further limiting our search within that group to those that investigated *isolated* cardiac conditions would have reduced our search to 17 articles for CAD (encompassing myocardial infarction, CABG, PCI), 5 articles for heart failure, and 2 articles for atrial fibrillation. We acknowledge that program length, intensity and cardiac disease type can all influence the physiological responses to cardiac rehabilitation; however, this review was necessary to determine the most appropriate direction to take for subsequent investigations and to highlight strengths and weaknesses in the current body of literature.

Sex differences that may be present after completion of a short CR program may not be evident with longer programs, or vice-versa. Indeed, CR programs longer than the typical 12 weeks have been shown to elicit further improvements [99]. Similarly, we did not extract data on daily physical activity which could play an important role in the physiological response to CR if it changed over the course of the program. A recent umbrella review found that in CR participants, physical activity increased and sedentary behavior decreased compared to with usual care [100]. Furthermore, some of the articles in the current review that proposed a physiological sex difference used populations with only HF or only atrial fibrillation, as compared to a combination of patients with CABG, CAD, history of MI, and/or angina. As all these conditions have different pathophysiology, it is certainly plausible that different HD populations would respond differently to CR or exercise. Another limitation could be that most HD patients are middle-aged and older and are likely to have passed menopause and progressed through andropause. Thus, the effects of sex hormones have been minimized, making men and women potentially less distinct in their responses to exercise training. Lastly, psychosocial factors were beyond the scope of this review but could also impede the improvement of physiological responses after CR by influencing such factors as program adherence (included in this review). Psychosocial factors that can affect adherence include patient motivation, understanding and awareness of the benefits, social support, and access to resources. These factors are particularly important to consider when investigating sex differences, as it has been shown that men and women have differing limitations to CR enrollment and adherence [101]. Though the psychosocial factors of adherence were not explored in this review, it is imperative to note that optimizing adherence can substantially enhance the success of cardiac rehabilitation programs and ultimately improve patient outcomes and impact comparisons.

The results of this systematic review were not quantitatively pooled in a meta-analysis due to the breadth of inclusion criteria. Therefore, sensitivity analyses and statistical power could not be assessed. Future reviews and analyses should build on the findings of this review by narrowing the inclusion criteria to draw more specific conclusions. An essential factor that could play a role in the outcomes observed is the type of statistical analysis conducted in each study. In this review, repeated measures ANOVAs, t-tests, and t-tests between deltas (i.e. change from CR) were the most frequently used statistics. In the ideal case of comparing the differences between the two sexes at two different time points, repeated measures ANOVAs should be conducted on disaggregated data to obtain the most accurate conclusions. Unfortunately, most of the studies in this current review used t-tests over time within each sex, preventing valid sex comparisons. Thus, due to the possibility that t-tests may reflect an inaccuracy of the results, many of our assessments where only one sex responded to CR may have been better interpreted as a greater response in one sex should the appropriate statistics have been conducted. We recommend that investigators consult statistical experts to ensure accurate conclusions when comparing sexes.

## Conclusions

Based on the findings of this systematic review without meta-analysis, men and women with HD respond similarly to CR. However, many articles have found larger improvements in men compared to women with regard to maximal oxygen consumption, functional capacity, 6MWD and grip strength. Reasons for such findings could be physiologically attributed to larger muscle mass in men compared to women. Though many other responses to CR have weakly suggested a sex difference, our work highlights that limitations such as low recruitment of women, statistics used, intervention type, type of HD population, adherence rates, and program length could dampen the ability to discern sex differences after CR. More research is required on CR effectiveness that includes larger cohorts of women and proper statistical analysis. It is important to note that our findings show that women benefit as much as men in most physiological measures highlighting that women should be prescribed CR as a standard therapy following a cardiac diagnosis.

### Electronic supplementary material

Below is the link to the electronic supplementary material.

Supplementary Material 1

Supplementary Material 2

## Data Availability

Data sharing is not applicable to this article as no datasets were generated or analyzed during the current study.
