# Supplementary material for: Sex differences in the physiological responses to cardiac rehabilitation: a systematic review
Source: BMC Sports Sci Med Rehabil. 2024 Mar 28;16:74. doi: 10.1186/s13102-024-00867-9 (PMC10976702; doi:10.1186/s13102-024-00867-9)
Supplement: Supplementary file 1 — Supplementary Material 1 [file 13102_2024_867_MOESM1_ESM.doc]

Supplementary Figure 1: Medline search strategy

**Heart Diseases, Cardiac Rehabilitatioin, & Gender Differences**

Search Strategy: Ovid MEDLINE(R) ALL <1946 to December 19, 2023>

--------------------------------------------------------------------------------

1 [Population: Heart Patients]

2 exp Heart Diseases/ (1224941)

3 ((cardiac or heart or coronary or myocardi*) adj3 (disease* or disorder* or anomol* or disturbance* or deficien* or deformit* or dysfunction* or ischemi* or patient*)).tw,kw. (559675)

4 ((rehab* or intervention*) adj3 (cardiac or cardiovascular or heart or coronary or infarct* or myocardi*)).tw,kw. (63119)

5 or/2-4 (1423231)

6 [Intervention: Cardiac/Exercise Rehab]

7 Cardiac Rehabilitation/ (3374)

8 exp Exercise Therapy/ (59593)

9 (exercis* adj3 (training or therap* or program* or rehab*)).tw,kw. (53808)

10 (training adj4 (resistanc* or weight* or strength* or interval* or intermittent* or cardio* or aerobic)).tw,kw. (33591)

11 (rehab* adj3 (cardiac or cardio* or program* or exercis*)).tw,kw. (32284)

12 or/7-11 (133098)

13 [Comparator: Gender]

14 Sex characteristics/ (59656)

15 sex factors/ (278026)

16 Sexism/ (2991)

17 gender*.tw,kw. (388470)

18 (sex* adj3 (differen* or compar* or between or impact* or barrier* or analys* or both or bias or disparit* or discriminat* or factor* or variable* or issue* or specific*)).tw,kw. (222890)

19 ((men or male) adj3 (compar* or versus or between or differen* or both) adj3 (women or female)).tw,kw. (71860)

20 or/14-19 (824628)

21 5 and 12 and 20 (1021)

22 limit 21 to "humans only (removes records about animals)" (1007)

***************************
