# Supplementary material for: Sex differences in the physiological responses to cardiac rehabilitation: a systematic review
Source: BMC Sports Sci Med Rehabil. 2024 Mar 28;16:74. doi: 10.1186/s13102-024-00867-9 (PMC10976702; doi:10.1186/s13102-024-00867-9)
Supplement: Supplementary file 2 — Supplementary Material 2 [file 13102_2024_867_MOESM2_ESM.docx]

# Supplemental Table 1: Additional Physiological Response Comparisons (only ≤2 article per investigated variable)

| **Physiological Response** | | **Greater improvement in men** | | **Only men improved** | **Greater improvement in women** | | **Only women improved** | | **Both men and women improved** | | **Neither sex improved** |
| --- | --- | --- | --- | --- | --- | --- | --- | --- | --- | --- | --- |
| **Fitness** | | | | | | | | | | | |
| Weekly training volume (MET/wk) | |  | |  |  | |  | | Calvo-Lopez 2023 | |  |
| VT1 (MET) | |  | | Calvo-Lopez 2023 |  | |  | |  | |  |
| VT2 (MET) | |  | | Calvo-Lopez 2023 |  | |  | |  | |  |
| VO_2_ relative (ml/kg/min) | |  | | Keteyian 2003 |  | |  | |  | |  |
| VO_2_/LBM @ VT1  (ml/min x kg) | |  | |  |  | |  | |  | | Trachsel 2020 |
| VO_2_/LBM at VT2  (ml/min x kg) | |  | |  |  | |  | |  | | Trachsel 2020 |
| Stress test level | |  | |  |  | |  | | Stojanovic 2023 | |  |
| Treadmill velocity (km/hr) | |  | |  |  | |  | | Soleimani 2009 | |  |
| Peak Power Output (W) | |  | |  |  | |  | | Szmigielska 2022, Temfemo 2011 | |  |
| Peak power (% change) | |  | |  |  | |  | | Mertens 1996 | |  |
| Oxygen uptake efficiency slope | |  | |  |  | |  | |  | | Trachsel 2020 |
| VO_2_/Workload slope | |  | |  |  | |  | |  | | Trachsel 2020 |
| O_2_ Pulse (mL/beat) | |  | |  |  | |  | |  | | Trachsel 2020 |
| Maximum HR (%) | | Cannistra 1992 | |  |  | |  | |  | |  |
| Post-exercise HR (bpm) | |  | |  |  | |  | | Soleimani-Netajian 2009 | |  |
| HRR in the first min (bpm) | |  | |  |  | |  | | Cheragi 2021 | | Korzeniowska-Kubacka 2015 |
| HRR in the second min (bpm) | |  | |  |  | |  | | Cheragi 2021 | |  |
| Changes in HRR in the second minute (%) | | Korzeniowska-Kubacka 2017 | |  |  | |  | |  | |  |
| Submaximal Rate Pressure Product (bpm x mmHg) | |  | |  |  | |  | | Casey 2009 | | Ades 1992 |
| Submaximal HR (bpm) | |  | |  |  | |  | | Stojanovic 2023 | | Ades 1992 |
| Submaximal SBP (mmHg) | |  | |  |  | |  | |  | | Ades 1992 |
| Peak SBP (mmHg) | |  | |  |  | |  | |  | | Lavie 1995, Calvo-Lopez 2023 |
| SBP effort (mmHg) | |  | | Korzeniowska-Kubacka 2015 |  | |  | |  | |  |
| DBP effort (mmHg) | | Korzeniowska-Kubacka 2017 | |  |  | |  | |  | | Korzeniowska-Kubacka 2015 |
| DBP peak (mmHg) | |  | |  |  | |  | |  | | Calvo-Lopez 2023 |
| Physical activity (min/week) | | Turk-Adawi 2016 | |  |  | |  | | Jafri 2023 | |  |
| Relative energy expenditure (kcal/kg/wk) | |  | |  |  | |  | | Schultz 2010 | |  |
| Flexibility (cm) | |  | | Kim 2019 |  | |  | |  | |  |
| Balance (s) | |  | |  |  | |  | | Kim 2019 | |  |
| Horizontal squat (number) | |  | |  |  | |  | | Adams 1999 | |  |
| Shoulder press (% change) | |  | |  |  | |  | | Swank 2010 | |  |
| Latissimus pulldown (% change) | |  | |  |  | |  | | Swank 2010 | |  |
| Bicep curl (% change) | |  | |  |  | |  | | Swank 2010 | |  |
| Arm curl (# repetitions) | |  | |  |  | |  | | Calvo-Lopez 2023 | |  |
| Triceps extension (% change) | |  | |  |  | |  | | Swank 2010 | |  |
| Timed up and go (s) | |  | |  |  | |  | | Kim 2019 | |  |
| Gait velocity (m/s) | | Tyni-Lenne 1998 | |  |  | |  | |  | |  |
| HR in gait (bpm) | | Tyni-Lenne 1998 | |  |  | |  | |  | |  |
| Post-6MWT DBP (mm Hg) | |  | |  | Araya-Ramirez 2021- | |  | |  | |  |
| Post-6MWT SBP (mm Hg) | |  | |  | Araya-Ramirez 2021 | |  | |  | |  |
| Post-6MWT HR (bpm) | |  | |  |  | |  | | Araya-Ramirez 2021 | |  |
| HR at equal workloads (bpm) | |  | |  |  | |  | | Ocallaghan 1984 | |  |
| Functional Reach (cm) | |  | | Kim 2019 |  | |  | | Adams 1999 | |  |
| Leg press (number) | |  | |  |  | |  | | Adams 1999, Swank 2010 | |  |
| Leg extension (number) | |  | |  |  | |  | | Adams 1999, Swank 2010 | |  |
| **Metabolic Measures** | | | | | | | | | | | |
| Insulin (mg/dl) | |  | |  |  | |  | |  | | Brochu 2000, Thorin-Trescases 2016 |
| FBF (ml/min/100 Ml tissue) | |  | |  |  | |  | | Antunes-Correa 2010 | |  |
| FVR (U) | |  | |  |  | |  | | Antunes-Correa 2010 | |  |
| TGs: HDL | |  | |  |  | |  | |  | | Thorin-Trescases 2016 |
| MAP (mm Hg) | |  | |  |  | |  | |  | | Antunes-Correa 2010 |
| eGFR (mL/min/1.73m2) | |  | |  |  | |  | |  | | Kitagaki 2022 |
| Hemoglobin (g/dL) | |  | |  |  | |  | |  | | Kitagaki 2022 |
| **Body Composition** | | | | | | | | | | | |
| Trunk fat mass (%) | |  | |  |  | |  | | Thorin-Trescases 2016 | |  |
| Hip circumference (cm) | | Brochu 2000 | |  |  | |  | | Sarrafzadegan 2008 | |  |
| LBM (kg) | |  | |  |  | |  | | McConnell 1997 | |  |
| BCM (%) | | Socha 2017 | |  |  | |  | |  | |  |
| TBW (%) | |  | |  |  | |  | | Socha 2017 | |  |
| Waist-to-hip ratio | |  | | Brochu 2000 |  | |  | | Sarrafzadegan 2008 | |  |
| Fat-free mass (%) | |  | | Brochu 2000 |  | |  | | Socha 2017 | |  |
| **Respiratory** | | | | | | | | | | | |
| Forced Vital Capacity (L) | |  | |  |  | |  | | Temfemo 2011 | | Ades 1992 |
| VE/VCO_2_ slope | |  | |  |  | |  | | Antunes-Correa 2010 | | Trachsel 2020 |
| VE at peak (L/min) | |  | |  |  | |  | |  | | Ades 1992, Trachsel 2020 |
| Breathing Frequency (breaths/min) | |  | |  |  | |  | |  | | Trachsel 2020 |
| Tidal volume (L) | |  | |  |  | |  | |  | | Trachsel 2020 |
| VE (L/min) | |  | |  |  | |  | |  | | Ades 1992 |
| Ventilatory threshold (ml/kg/min) | |  | |  |  | |  | | Temfemo 2011 | |  |
| Peak Expiratory flow (L) | |  | |  |  | |  | |  | | Temfemo 2011 |
| Submaximal exercise VE (L/min) | |  | |  |  | |  | |  | | Ades 1992 |
| FEV/FVC (%) | |  | |  |  | |  | | Temfemo 2011 | |  |
| Forced expiratory volume in 1 second (L/s) | |  | |  |  | |  | | Temfemo 2011 | |  |
| **Cardiac Measures** | | | | | | | | | | | |
| NYHA class | |  | |  |  | |  | | Antunes-Correa 2010 | |  |
| EF <50% (n values) | |  | |  |  | |  | | Pabisiak 2013 | |  |
| Changes in Chronotropic index (%) | |  | | Anjo 2014 |  | |  | |  | |  |
| LVMI (g/m^2^) | |  | |  |  | |  | |  | | Trachsel 2020 |
| LVEDVI (ml/m^2^) | |  | |  |  | |  | |  | | Trachsel 2020 |
| LVESV (ml) | |  | |  |  | |  | |  | | Trachsel 2020 |
| LVESVi (ml/m^2^) | |  | |  |  | |  | |  | | Trachsel 2020 |
| LVEDD (mm) | |  | |  |  | |  | |  | | El Missiri 2020, Trachsel 2020 |
| LVESD (mm) | |  | |  |  | |  | | El Missiri 2020 | | Trachsel 2020 |
| E/A ratio | |  | |  |  | |  | |  | | Trachsel 2020 |
| Early to late diastolic filling velocity | |  | |  |  | |  | |  | | Trachsel 2020 |
| Mean peak early diastolic mitral annulus velocity | |  | |  |  | |  | |  | | Trachsel 2020 |
| LA diameter (mm) | |  | |  |  | |  | |  | | Trachsel 2020 |
| Stroke volume index | |  | |  |  | |  | |  | | Trachsel 2020 |
| Cardiac Index | |  | |  |  | |  | |  | | Trachsel 2020 |
| **Muscle Physiology** |  | |  | | |  | |  | |  | |
| MHC-1 (% Change) | |  | | Keteyian 2003 |  | |  | |  | |  |
| MHC- 2a (% Change) | |  | |  |  | |  | |  | | Keteyian 2003 |
| MHC-2x (% Change) | |  | |  |  | |  | |  | | Keteyian 2003 |
| Capillary density (endothelial/muscle fiber) | |  | |  |  | |  | |  | | Keteyian 2003 |
| Blood lactate baseline (nmol/L) | |  | |  |  | |  | | Tyni-Lenne 1998 | |  |
| Blood lactate at 15 min point (nmol/L) | |  | |  |  | |  | | Tyni-Lenne 1998 | |  |
| **Autonomic** | | | | | | | | | | | |
| MSNA frequency (bursts/min) | |  | |  |  | |  | | Antunes-Correa 2010 | |  |
| MSNA incidence (bursts/100 heart beats) | |  | |  |  | |  | | Antunes-Correa 2010 | |  |
| Plasma Norepinephrine baseline (nmol/L) | |  | |  |  | |  | | Tyni-Lenne 1998 | |  |
| Plasma Norepinephrine 15 min point (nmol/L) | |  | |  |  | |  | | Tyni-Lenne 1998 | |  |
| 24hr SBP variability (mmHg) | |  | |  |  | |  | | Caminiti 2022 | |  |
| Daytime SBP variability (mmHg) | |  | |  |  | | Caminiti 2022 | |  | |  |
| Nighttime SBP variability (mmHg) | |  | |  |  | |  | |  | | Caminiti 2022 |
| 24hr DBP variability (mmHg) | |  | | Caminiti 2022 |  | |  | |  | |  |
| Daytime DBP variability (mmHg) | |  | |  |  | | Caminiti 2022 | |  | |  |
| Nighttime DBP variability (mmHg) | |  | |  | Caminiti 2022 | |  | |  | |  |
| 24hr SBP (mmHg) | |  | | Caminiti 2022 |  | |  | |  | |  |
| Daytime SBP (mmHg) | |  | | Caminiti 2022 |  | |  | |  | |  |
| Nighttime SBP (mmHg) | |  | | Caminiti 2022 |  | |  | |  | |  |
| 24hr DBP (mmHg) | |  | | Caminiti 2022 |  | |  | |  | |  |
| Daytime DBP (mmHg) | |  | |  |  | |  | | Caminiti 2022 | |  |
| Nighttime DBP (mmHg) | |  | | Caminiti 2022 |  | |  | |  | |  |
| 24hr HR (bpm) | |  | |  |  | |  | |  | | Caminiti 2022 |
| Daytime HR (bpm) | |  | |  |  | |  | |  | | Caminiti 2022 |
| Nighttime HR (bpm) | |  | |  |  | |  | | Caminiti 2022 | |  |
| **Protein Measures** | | | | | | | | | | | |
| Paraoxonase (μm/ml) | |  | |  |  | |  | | Goldhammer 2007 | |  |
| p-selectin expression (%) | |  | |  |  | | Keating 2013 | |  | |  |
| BNP (ng/L) | | Sheikhian 2018 | |  |  | |  | |  | |  |
| NT-proBNP (pg/ml) | |  | |  | Anjo 2014 | |  | | Kitagaki 2022 | |  |
| Angptl2 (ng/ml) | |  | | Thorin-Trescases 2016 |  | |  | |  | |  |
| Cytochrome C Oxidase Change (mol/protein x h) | |  | |  |  | |  | | Keteyian 2003 | |  |
| HAD Change (mol/kg protein x h) | |  | |  |  | |  | | Keteyian 2003 | |  |
| Citrate Synthase Change (mol/ protein x h) | |  | |  |  | |  | | Keteyian 2003 | |  |
| Trimethlyamine n-oxide | |  | |  |  | | Baranyi 2022 | |  | |  |

6MWT; 6 minute walk time, BCM; body cell mass, BF; breathing frequency, BNP; brain natriuretic peptide, DBP; diastolic blood pressure, E/A; early filling to late diastolic filling ratio, EF; ejection fraction, eGFR; estimated glomerular filtration rate, FBF; forearm blood flow, FVR; forearm vascular resistance, FEV/FVC; forced expiratory volume/forced vital capacity, HAD: haloacid dehydrogenase, HDL; high density lipoprotein, HR; heart rate, HRR; heart rate recovery, LA diameter; left atrium diameter, LBM; lean body mass, LVEDD; left ventricular end diastolic diameter; LVEDVI; left ventricular end diastolic volume index, LVESD; left ventricular end systolic diameter, LVESV; left ventricular end systolic volume, LVESVI; left ventricular end systolic volume index, LVMI: left ventricular mass index, MAP; mean arterial pressure, MET; metabolic equivalent, MHC; myosin heavy chain form, MSNA; muscle sympathetic nerve activity, NT-proBNP: N-terminal pro brain natriuretic peptide, NYHA; New York Heart Association, SBP; systolic blood pressure, TG; triglyceride, TBW: total body water, VE; ventilation, VO2; volume of oxygen consumption, VT1 and VT2; first and secondary ventilatory threshold
